# Integrated stress response of vertebrates is regulated by four eIF2α kinases

**DOI:** 10.1038/srep32886

**Published:** 2016-09-16

**Authors:** Shusuke Taniuchi, Masato Miyake, Kazue Tsugawa, Miho Oyadomari, Seiichi Oyadomari

**Affiliations:** 1 Division of Molecular Biology, Institute for Genome Research, Institute of Advanced Medical Sciences, Tokushima University, Tokushima 770-8503, Japan; 2 Department of Molecular Research, Diabetes Therapeutics and Research Center, Institute of Advanced Medical Sciences, Tokushima University, Tokushima 770-8503, Japan; 3 Fujii Memorial Institute of Medical Sciences, Institute of Advanced Medical Sciences, Tokushima University, Tokushima 770-8503, Japan

## Abstract

The integrated stress response (ISR) is a cytoprotective pathway initiated upon phosphorylation of the eukaryotic translation initiation factor 2 (eIF2α) residue designated serine-51, which is critical for translational control in response to various stress conditions. Four eIF2α kinases, namely heme-regulated inhibitor (HRI), protein kinase R (PKR), PKR-like endoplasmic reticulum kinase, (PERK) and general control non-depressible 2 (GCN2), have been identified thus far, and they are known to be activated by heme depletion, viral infection, endoplasmic reticulum stress, and amino acid starvation, respectively. Because eIF2α is phosphorylated under various stress conditions, the existence of an additional eIF2α kinase has been suggested. To validate the existence of the unidentified eIF2α kinase, we constructed an eIF2α kinase quadruple knockout cells (4KO cells) in which the four known eIF2α kinase genes were deleted using the CRISPR/Cas9-mediated genome editing. Phosphorylation of eIF2α was completely abolished in the 4KO cells by various stress stimulations. Our data suggests that the four known eIF2α kinases are sufficient for ISR and that there are no additional eIF2α kinases in vertebrates.

The α subunit of eukaryotic translation initiation factor 2 (eIF2α) is known to be phosphorylated by diverse stress stimuli^1^. There are several phosphorylation sites in eIF2α, but serine 51 is critical for translational control^2^. eIF2α phosphorylation causes transient attenuation of the translation of most mRNAs and transcriptional induction by selective translation of transcription factors. These signalling programs allow cells to adapt to various stress conditions, referred to as the integrated stress response (ISR)^3^,^4^. Recent studies have shown that ISR plays physiological roles in the regulation of intermediary metabolism^5^,^6^, memory^7^,^8^, tumorigenesis^9^, and immunity^10^. Therefore, it is important to understand the mechanism in which the physiological or pathological stimuli are coupled to eIF2α phosphorylation.

Four distinct eIF2α kinases have been identified in vertebrates. Each kinase is activated by stimulatory factors that bind to its regulatory domains, which promote the active state dimer configuration of its catalytic kinase domains. For example, heme-regulated inhibitor (HRI/EIF2AK1) is activated during heme deficiency by the release of heme from its kinase insert domains^11^,^12^. Protein kinase R (PKR/EIF2AK2) is activated during a viral infection by the binding of double-stranded RNA (dsRNA) to its two conserved dsRNA binding domains^13^,^14^. PKR-like endoplasmic reticulum (ER) kinase (PERK/EIF2AK3) is activated during ER stress by the release of binding immunoglobulin protein from its ER luminal domains^15^,^16^. General control non-depressible 2 (GCN2/EIF2AK4) is activated under amino acid deprivation by the binding of uncharged tRNA to the regulatory domains^17^,^18^. Although much is known about the structural and functional features of these four eIF2α kinases, there are key questions that remain to be elucidated, such as what eIF2α kinases are responsible for certain stress stimuli and are there additional eIF2α kinases that have not been identified. Therefore, the primary aim of the present study was to validate the existence of additional eIF2α kinase in vertebrates.

We observed 14 types of diverse stress stimuli that induced eIF2α phosphorylation. Surprisingly, this eIF2α phosphorylation was completely abolished in quadruple knockout cells (4KO cells) for all four known eIF2α kinases, suggesting that the four known eIF2α kinases are sufficient for ISR and that there are no additional eIF2α kinases in vertebrates.

## Results

### Possibility of additional eIF2α kinases among eukaryotes

**Figure 1:**
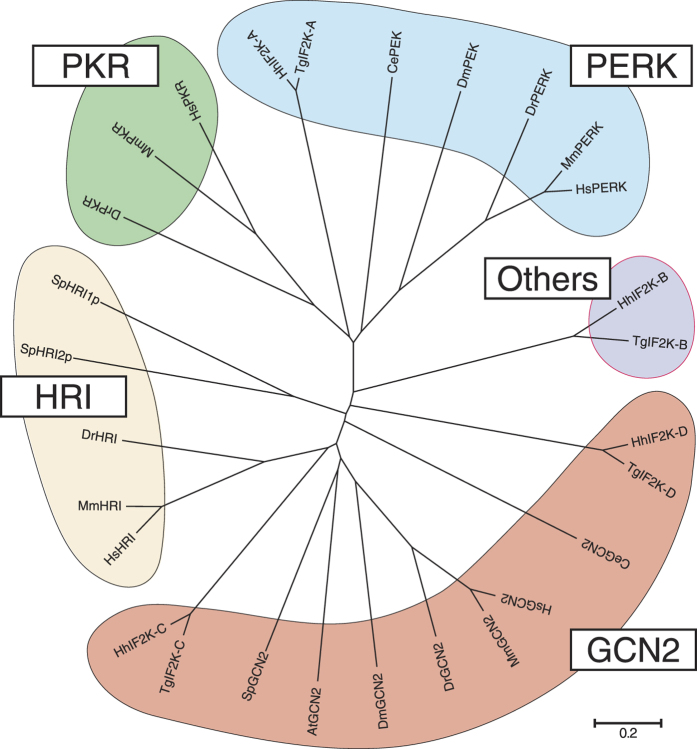
Phylogenetic analysis of the eukaryotic translation initiation factor 2 (eIF2α) kinomes. The amino acid sequences of kinases that are homologous to eIF2α kinases were aligned using CLUSTAL W 2.1 and the unrooted phylogenetic tree was created via the neighbour-joining method using MEGA 5.05. The scale bar represents 0.2 substitutions per site. The species abbreviations used in the Figure were as follows: *Ha Hammondia hammondi* (Hh), *Toxoplasma gondii* (Tg), *Arabidopsis thaliana* (At), *Schizosaccharomyces pombe* (Sp), *Caenorhabditis elegans* (Ce), *Drosophila melanogaster*, *Danio rerio* (Dr) and *Mus musculus* (Mm).

The completion of genomic sequencing has permitted classifications and comparisons with model organism kinomes, which has identified putative protein kinase genes. Kinases were classified into 10 groups primarily by sequence comparison of their catalytic domains, aided by knowledge of the biological functions^19^,^20^. The four known eIF2α kinases, namely as HRI, PKR, PERK, and GCN2, are categorized as eukaryotic protein kinases (ePKs), but they do not fit into any of the 10 ePK groups. Therefore, these eIF2α kinases are categorized into the other group and no criterion for classifying the eIF2α kinases has been established. The classification has grown as new subfamilies have been added for kinases that are conserved among multiple organisms. To gain insights into additional eIF2α kinases, phylogenetic comparison of eIF2α kinomes among plants, yeast, parasites, worms, flies, fish, mice, and humans was conducted (Fig. 1). Kinases were first clustered as a family by sequence similarity in the kinase domain and additional information from subcellular localizations and known functions.

GCN2 is conserved throughout eukaryotes from plants such as *Arabidopsis thaliana* to animals. HRI is conserved in fungi, such as *Schizosaccharomyces pombe*, and many vertebrates from *Danio rerio* to *Homo sapiens*, although some exceptions have been observed, such as *Drosophila melanogaster* and *Caenorhabditis elegans*. However, it is not found in plants or protists, such as *Hammondia hammondi* and *Toxoplasma gondii.* PKR is only conserved in vertebrates, whereas it is not found in plants, fungi, protists, and invertebrates. PERK is conserved in protists, invertebrates, and vertebrates but not in plants and fungi. Interestingly, newly identified protist eIF2α kinases^21^,^22^,^23^ formed a fifth eIF2α kinase cluster independent of the other four kinases. The kinase classification remains imperfect and incomplete. Indeed, several new subfamilies have been added for kinases that are conserved among multiple phyla. Therefore, we hypothesized that an additional kinase may exist in vertebrates.

### Various stress conditions cause ISR

**Figure 2:**
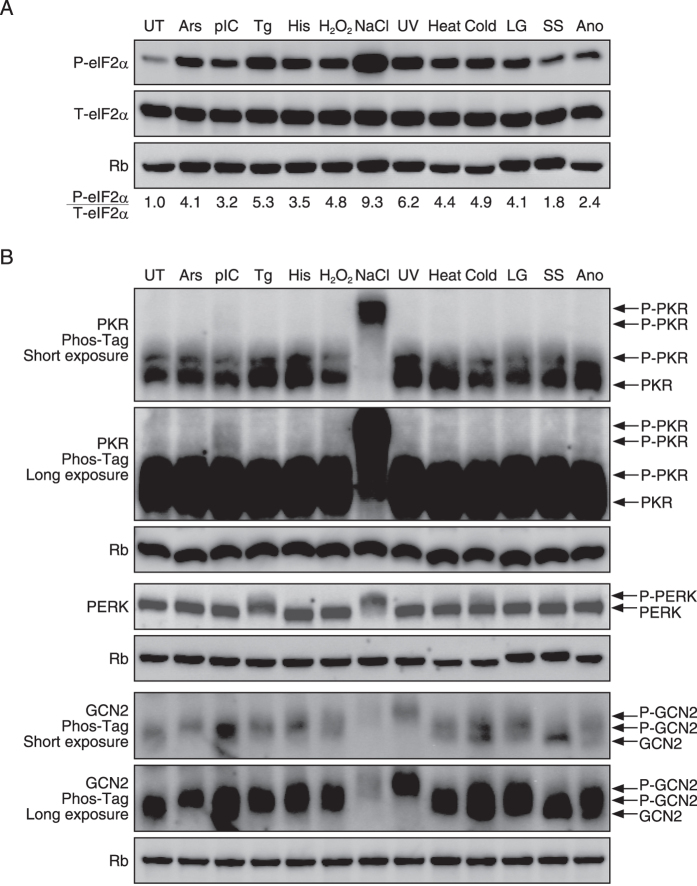
Eukaryotic translation initiation factor 2 (eIF2α) is phosphorylated by various stresses in mouse embryonic fibroblasts (MEFs). (**A**) Representative immunoblots of phosphorylated eIF2α, total eIF2α and ribophorin (Rb) 1 h after treatment with the indicated stress stimulus in the wild-type MEFs. The ratio of autophosphorylated versus total eIF2α is indicated. (**B**) Representative immunoblots of phosphorylated PKR, PERK, GCN2 and Rb 1 h after treatment with the indicated stress stimulus in MEFs. The stress condition abbreviations used in the Figure were as follows: untreated (UT), 400 μM arsenate (Ars), 10 ng/μl polyinosinic-polycytidylic acid (pIC), 200 nM thapsigargin (Tg), 2 mM histidinol (His), 1 mM H_2_O_2_ (H_2_O_2_), 500 mM NaCl (NaCl), 254 nm 200 J/m^2^ ultraviolet irradiation (UV), 42 °C heat shock (heat), cold shock (cold), low glucose (LG), serum starvation (SS) and anoxia (Ano).

The eIF2α kinases are known to be activated primarily by specific stressors. Hence, to validate the existence of additional eIF2α kinases, it is important to know what types of stressors can activate ISR. Mouse embryonic fibroblasts (MEFs) treated with 12 distinct stressors for 1 h were concomitantly analysed for the phosphorylation of eIF2α (Fig. 2A). We found that all 12 distinct stressors phosphorylated eIF2α (Fig. 2A). The degree of eIF2α phosphorylation was quantified by the ratio of phosphorylated/non-phosphorylated eIF2α. NaCl, ultraviolet light (UV), and thapsigargin (Tg) caused strong eIF2α phosphorylation; cold shock (Cold), H_2_O_2_, heat shock (Heat), low glucose (LG), arsenate (Ars), and histidinol (His) caused moderate phosphorylation; and polyinosinic-polycytidylic acid (pIC), anoxia (Ano), and serum starvation (SS) caused mild phosphorylation (Fig. 2A).

The expression of three eIF2α kinases (PKR, PERK, and GCN2) (Fig. 2B) was assessed by immunoblot analysis. Because none of the two antibodies against HRI that we purchased could recognize mouse HRI under our conditions, we could not evaluate the phosphorylation of HRI. The activation of PKR by an arsenic compound in mammalian cells was described in an earlier paper^24^, but this activation was refuted by other groups using PKR knockout cells, which reported the activation of HRI by an arsenic compound^25^,^26^. In contrast, a report was published stating that an arsenic compound activated HRI and GCN2 in yeast^27^. When we treated MEFs with 400 μM Ars, the phosphorylation of eIF2α and GCN2 was observed, but the phosphorylation of PKR or PERK was not detected by a phosphorylation-dependent mobility shift assay on standard or phos-tag SDS-PAGE gel (Fig. 2A,B). Thus, it was suggested that Ars activated HRI and GCN2 in accordance with previous reports. A synthetic analog of dsRNA, polyinosinic-polycytidylic acid (polyIC), mimics RNA viral infection, which is known to activate PKR^28^. Tg is an inhibitor of the sarcoplasmic/endoplasmic reticulum Ca^2+^ ATPase, which is known to induce ER stress and PERK activation^15^. The histidine analog histidinol (His) inhibits histidyl-tRNA synthetase activity, which is known to increase uncharged tRNA levels and activate GCN2^29^. In agreement with previous reports, MEFs treated with 10 ng/μl polyIC, 200 nM Tg, or 2 mM histidinol displayed increased phosphorylation of eIF2α and activation of PKR, PERK, and GCN2, respectively (Fig. 2A,B).

We examined the activation of eIF2α kinases by H_2_O_2_, UV, heat shock, cold shock, low glucose, serum starvation, and anoxia, for which it was not obvious which eIF2α kinase was activated. We found that 1 mM H_2_O_2_ induced phosphorylation of eIF2α and GCN2, but phosphorylation of PKR or PERK was not observed (Fig. 2A,B). As H_2_O_2_ has been reported to activate HRI in *S. pombe*^27^, H_2_O_2_ can possibly activate two eIF2α kinases, GCN2 and HRI. Under hyperosmotic stress induced by NaCl, phosphorylation of eIF2α via HRI has been reported but not the involvement of any other kinases^25^. We observed strong phosphorylation of eIF2α by 500 mM NaCl, in line with previous reports, and activation of PKR, PERK and GCN2 (Fig. 2A,B and Supplemental Fig. 1). UV irradiation has been reported to activate PERK^30^ and GCN2^31^. We found that exposure to UV (254 nM) induced strong phosphorylation of eIF2α and activated GCN2 as previously reported but not PERK nor PKR (Fig. 2A,B). It has been reported that heat shock induces eIF2α via HRI but not any other kinases^25^. We observed weak phosphorylation of eIF2α in response to heat shock at 42 °C, which did not induce the activation of PKR, but weak activation of PERK and GCN2 was noted (Fig. 2A,B). Upon cold shock on ice, we observed phosphorylation of eIF2α, activation of PERK, as previously reported^32^, and weak activation of GCN2 and PKR. Low glucose (1 g/l glucose) induced phosphorylation of eIF2α, but it did not induce PERK activation, which was previously reported^33^,^34^ (Fig. 2A,B). Low glucose did not activate PKR, but it was found to activate GCN2 (Fig. 2A,B). Serum starvation induced weak phosphorylation of eIF2α, GCN2 activation, as previously reported^35^,^36^, and weak activation of PERK, but it did not induce PKR activation (Fig. 2A,B). Anoxia induced weak phosphorylation of eIF2α and activation of PERK and GCN2, as previously reported^37^,^38^, but it did not induce PKR activation (Fig. 2A,B). From the results, it was verified that among various stress conditions that induce eIF2α phosphorylation, there are stresses that specifically activate one of the known eIF2α kinases and those that activate two or more kinases. However, our experiments were conducted at a specific concentration of stress agent, a specific time point, and the analysis was in a specific cell type: MEF cells. Therefore, there are many variables that can account for the differences between the results presented in this paper and in other published reports.

### Establishment of a 4KO cell line lacking the four known kinases

**Figure 3:**
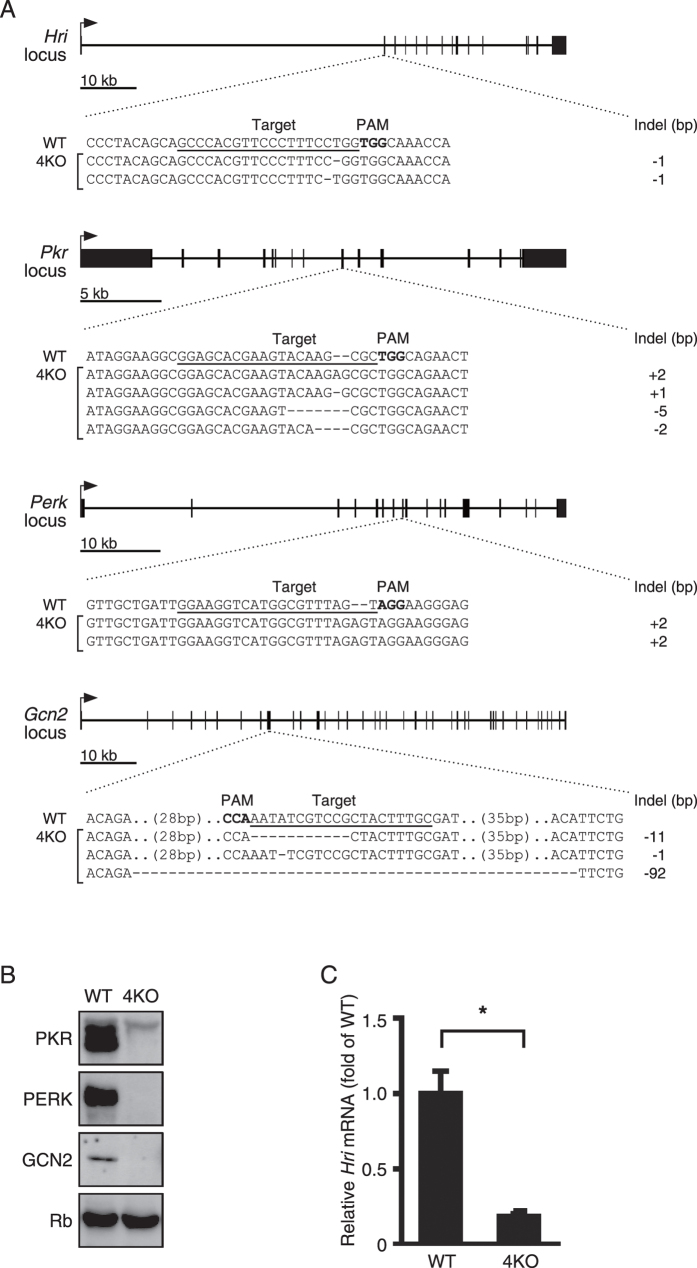
Generation of quadruple eukaryotic translation initiation factor 2 (eIF2α) kinase knockout mouse embryonic fibroblasts (MEFs) (4KO) by CRISPR/Cas9-mediated genome editing. (**A**) Schematic of each targeting locus of the four *Eif2ak* kinases with the target sequence underscored, PAM in bold type and exons in block boxes. Representative sequencing results showing the indel mutations generated in 4KO cells. (**B**) Representative immunoblots of protein kinase R (PKR), PKR-like ER kinase (PERK), general control non-depressible 2 (GCN2) and ribophorin (Rb) in the wild-type and 4KO MEFs. (**C**) RT-qPCR analysis (mean ± SD, n = 4, *p < 0.01) of the expression of heme-regulated inhibitor (*Hri*) mRNA in the wild-type and 4KO MEFs.

The recently developed genome engineering strategy using CRISPR/Cas9 systems^39^,^40^ enables two or more genes to be destroyed simultaneously, which has been a difficult, time-consuming, and laborious task. We established an MEF line that lacks all four eIF2α kinases using CRISPR/Cas9 technology. Upon DNA sequencing, we confirmed that the four gRNAs for the eIF2α kinases efficiently induced indel mutations in the respective genes in the 4KO cells (Fig. 3A and Supplemental Fig. 2). Via immunoblotting, we ascertained that PKR, PERK, and GCN2 were not expressed in the 4KO cells at the protein level (Fig. 3B). Since there was no antibody available that could detect endogenous HRI, we confirmed by reverse transcription-quantitative polymerase chain reaction (RT-qPCR) that the mRNA expression of the gene was remarkably low (Fig. 3C).

### There are no additional eIF2α kinases other than the known four kinases

**Figure 4:**
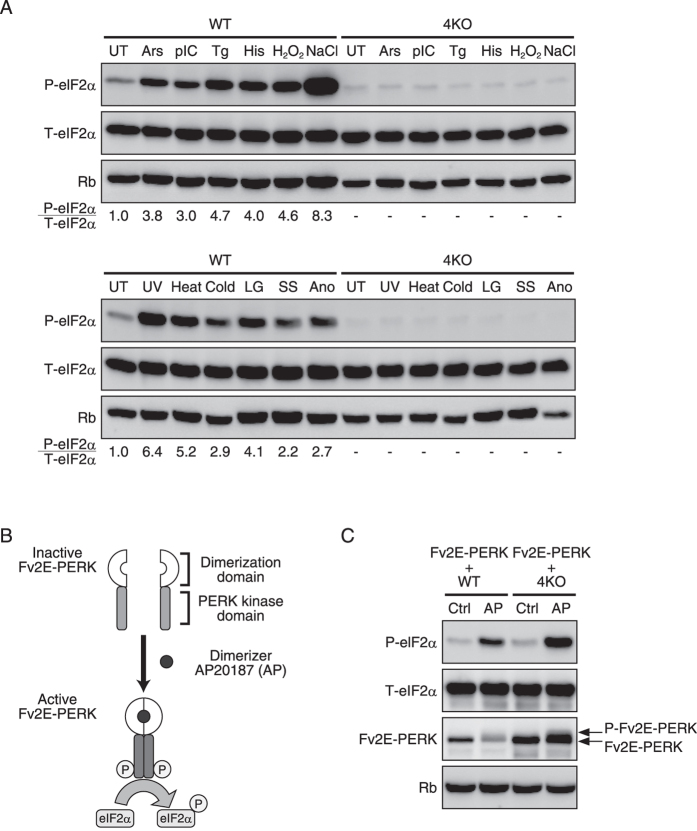
Eukaryotic translation initiation factor 2 (eIF2α) phosphorylation was completely abolished in quadruple eIF2α kinase knockout mouse embryonic fibroblasts (4KO MEFs) under the various stress conditions. (**A**) Representative immunoblots of phosphorylated eIF2α, total eIF2α and ribophorin (Rb) 1 h after treatment with the indicated stress stimulus in the wild-type and 4KO MEFs. The ratio of autophosphorylated versus total eIF2α is indicated. The stress conditions abbreviations used in the Figure are as follows: untreated (UT), 400 μM arsenate (Ars), 10 ng/μl polyIC (pIC), 200 nM thapsigargin (Tg), 2 mM histidinol (His), 1 mM H_2_O_2_ (H_2_O_2_), 500 mM NaCl (NaCl), 254 nm 200 J/m^2^ ultraviolet irradiation (UV), 42 °C heat shock (heat), cold shock (cold), low glucose (LG), serum starvation (SS) and anoxia (Ano). (**B**) Schematic representation of the structure and activation of the Fv2E-PERK fusion protein. AP20187(AP)-induced Fv2E-PERK homodimerization causes the activation of Fv2E-PERK, subsequently phosphorylating eIF2α. (**C**) Representative immunoblots of phosphorylated eIF2α, total eIF2α and Fv2E-PERK in the wild-type and 4KO MEFs overexpressing Fv2E-PERK after exposure to Mock (Ctrl) or 1 nM AP for 1 h.

As the phylogenetic tree suggests, phosphorylation of eIF2α should be observed in the established 4KO cells upon exposure to some type of stress condition if there is any additional eIF2α kinase other than the known four kinases. We applied twelve different stress stimuli that are known to induce phosphorylation of eIF2α to the established 4KO cells and analysed eIF2α phosphorylation. Against our hypothesis, phosphorylation of eIF2α completely disappeared for all twelve different stress stimuli (Fig. 4A). To verify that the disappearance of eIF2α phosphorylation was not attributable to genome engineering due to an off-target effect of the CRISPR/Cas9 systems, a ligand-activatable PERK kinase called Fv2E-PERK was overexpressed in the 4KO cells. Fv2E-PERK consists of a cytosolic PERK kinase domain and an artificial dimerization domain (Fv2E) (Fig. 4B). The application of the specific dimerizer AP20187 (AP) can activate its PERK kinase domain. AP-dependent phosphorylation of eIF2α was observed, denying the possibility that genome engineering was the cause of the disappearance of eIF2α phosphorylation in the 4KO cells (Fig. 4C). From these results, it can be deduced that there are no additional eIF2α kinases other than HRI, PKR, PERK, and GCN2 and that these four eIF2α kinases are entirely responsible for ISRs against various stress conditions.

### The 4KO cells represent an effective tool for identifying the kinase responsible for ISR activation

**Figure 5:**
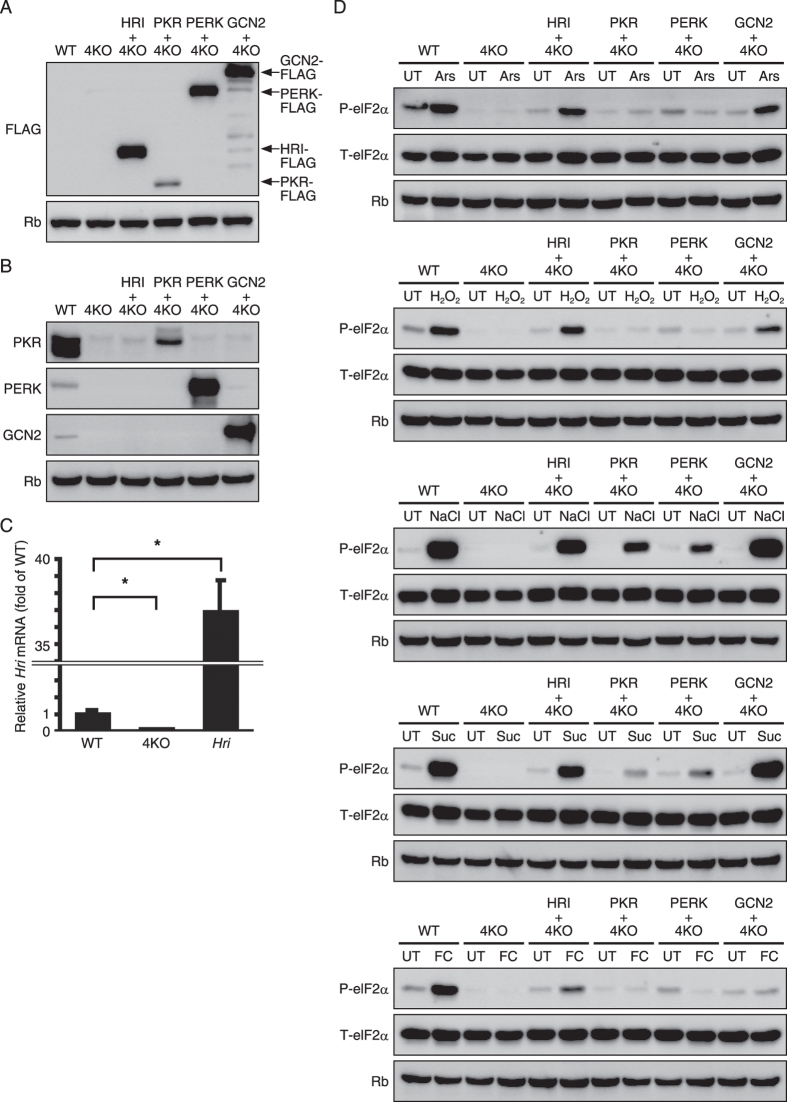
Quadruple eukaryotic translation initiation factor 2 (eIF2α) kinase knockout mouse embryonic fibroblasts (4KO MEFs) overexpressing the indicated Flag-tagged eIF2α kinases are useful tools for validating the activation of each eIF2α kinase by various stressors. (**A**) Representative immunoblots showing ectopically expression of the indicated Flag-tagged eIF2α kinases and endogenous ribophorin (Rb) in the six types of MEFs (wild-type, 4KO and 4KO overexpressing the indicated Flag-tagged eIF2α kinases). (**B**) Representative immunoblots of protein kinase R (PKR), PKR-like ER kinase (PERK), general control non-depressible 2 (GCN2) and Rb in the six types of MEFs (wild-type, 4KO and 4KO overexpressing the indicated Flag-tagged eIF2α kinases). (**C**) RT-qPCR analysis (mean ± SD, n = 4, *p < 0.01) of the mRNA expression of heme-regulated inhibitor (*Hri*) in the six types of MEFs (wild-type, 4KO and 4KO overexpressing the indicated Flag-tagged eIF2α kinases). (**D**) Representative immunoblots of phosphorylated eIF2α, total eIF2α and Rb 1 h after treatment with the indicated stress stimulus in the six types of MEFs (wild-type, 4KO and 4KO overexpressing the indicated Flag-tagged eIF2α kinases).

Cells are likely to acquire stress tolerance against various stress stimuli via the four eIF2α kinases responding in an ingenious overlapping manner. There is a possibility that a stress stimulus activates two or more eIF2α kinases. However, the magnitude of the activation of the primary kinase overshadows that of the secondary kinase, making the latter kinase difficult to detect. To overcome this problem, we established eIF2α kinase rescue 4KO cell lines in which a single eIF2α kinase was rescued. All eIF2α kinases that respond to a stress stimulus can be identified by applying the stimulus to the cell lines and observing the phosphorylation of eIF2α. In the 4KO cell lines, an eIF2α kinase carrying a FLAG tag was re-introduced using a retrovirus. By immunoblotting using FLAG antibody, we confirmed that the expression level of the eIF2α kinase in the four eIF2α kinase rescue 4KO cell lines was mutually similar (Fig. 5A). Via immunoblotting or RT-qPCR, we confirmed that the eIF2α kinases carrying the FLAG tag were overexpressed in the 4KO cells compared with the wild-type endogenous eIF2α kinases (Fig. 5B,C).

The involvement of ISR in oxidative, osmotic, and mitochondrial oxidative stresses, which has not been clarified, was investigated using the eIF2α kinase rescue 4KO cell lines (Fig. 5D). The phosphorylation of eIF2α in response to 400 μM Ars was recovered only when HRI and GCN2 were expressed, which was consistent with a previous finding in yeast^27^. Phosphorylation of eIF2α in response to 1 mM H_2_O_2_ was recovered only when HRI and GCN2 were expressed. Interestingly, we found that it is possible to recover eIF2α phosphorylation in response to 500 mM NaCl, which causes ionic and hyperosmotic stress, or 500 mM sucrose, which causes non-ionic and hyperosmotic stress, by rescuing any of the four eIF2α kinases. Furthermore, eIF2α phosphorylation induced by 25 μM FCCP, which blocks mitochondrial oxidative phosphorylation, could be recovered only by the expression of HRI (Fig. 5D). The 4KO cell lines established in this study were demonstrated to represent effective tools for identifying which eIF2α kinase is the primary or secondary kinase for activating ISR.

## Discussion

ISR is a highly conserved adaptation to stress. However, the type of eIF2α kinase differs among the kingdoms. In plants, GCN2 is the sole eIF2α kinase^41^. Invertebrates and fungi have two eIF2α kinases and protists and vertebrates carry four eIF2α kinases. Protists lack eIF2α kinases that correspond to HRI and PKR but have two eIF2α kinases in addition to the orthologs of GCN2 and PERK. This evidence suggests that the number of eIF2α kinases increased in the course of evolution among plants, fungi, and animals to permit proper responses to diverse stresses. Protists are assumed to have undergone a peculiar course of evolution to survive as a parasite in its metazoan host and acquired two types of eIF2α kinases that differ from those of other eukaryotes. The results of our analysis using 4KO cells derived from a vertebrate suggest that there is no eIF2α kinase universally present in the cells of vertebrates other than the four known kinases. This is in agreement with the disappearance of eIF2α phosphorylation, which was slightly observed in the 4KO cells under non-stressed conditions. However, as our experiments were conducted in a specific cell type (MEF cells), at a specific concentration of stress agents, and at a specific time point (1 hour), there is still the possibility that a cell-specific eIF2α kinase is present in vertebrates. As ISR is involved in the regulation of diverse biological functions, it is worth testing further stress conditions in a future study.

eIF2α kinases have a mutually similar catalytic domain. They have a common characteristic in which their phospho-transfer function is regulated by dimerization. The four kinases have mutually different regulatory domains and this likely explains why they can respond to different stress stimuli. On the contrary, it is interesting that oxidative and osmotic stresses activate two or more eIF2α kinases. This suggests the existence of a molecular mechanism that activates ligands other than the primary target ligand of activation. For example, heme is a natural negative regulator of HRI, and it inhibits the conformation change necessary for autophosphorylation by binding with the HRI dimer. Both nitric oxide (NO) and carbon monoxide (CO) bind to the N-terminal heme-binding domain of HRI. NO acts as an activator of HRI and CO serves as a suppressor of NO-induced HRI activation^42^. Similarly to these gases, a stimulus could possibly act directly on a regulatory domain and indirectly on a regulatory domain via a binding protein. For example, PACT (a protein activator) has been reported to bind to the dsRNA binding domain of PKR and upon phosphorylation, activate PKR in a process not associated with dsRNA^24^. For drug development by ISR regulation, it is essential to unveil the mechanisms by which various stress stimuli activate eIF2α kinases. ISR is likely to be involved in the regulation of diverse biological functions and the onset of diseases. Compounds that regulate eIF2α kinase activity, such as via HTS, have been discovered^43^, but it is important to understand eIF2α kinase selectivity of the compounds. Our developed 4KO cells and eIF2α kinase rescue 4KO cells, which express individual eIF2α kinases, are effective tools for accelerating studies on the molecular mechanism of eIF2α kinase activity.

## Methods

### Cell culture, transfection, and transduction

SV40 large T-antigen immortalized MEFs were cultured in DMEM-high glucose supplemented with 10% FBS, 2 mM l-glutamine, 55 μM 2-mercaptoethanol, and nonessential amino acids (Invitrogen). PlatGP cells were cultured in DMEM-high glucose supplemented with 10% FBS. Transfection was conducted using the Neon Transfection System (Invitrogen) at 1400 V and 30 ms or polyethylenimine (PEI) (Polysciences). Mouse *Hri*, *Pkr*, *Perk*, and *Gcn2* were cloned in-frame with a COOH-terminal 3X-FLAG into the retroviral expression vector pMXs-IG or pMXs-IP (kindly provided by Dr. T. Kitamura). Transfection for retrovirus production was performed using PEI. Retroviral transduction was performed using a previously published protocol with modifications^44^.

### Stress conditions

To validate the ISR activation, MEFs were treated for 1 hour with 400 μM arsenate (Ars), 10 ng/μl polyinosinic-polycytidylic acid (pIC), 200 nM thapsigargin (Tg), 2 mM histidinol (His), 1 mM H_2_O_2_ (H_2_O_2_), 500 mM NaCl (NaCl), 254 nm 200 J/m^2^ ultraviolet irradiation (UV), 42 °C heat shock (heat), 4 °C cold shock (cold), low glucose (LG), serum starvation (SS), and anoxia (Ano). Transfection with polyIC was performed using PEI. UV (254 nm) irradiation (200 J/m^2^) was performed in a CL-1000 UV cross-linker (UVP). Anoxia was induced by placing cells in am MIC-101 modular incubator chamber (Billups-Rothenberg Inc.) flushed with 100% nitrogen gas at 37 °C.

### Genome editing using the CRISPR-Cas9 system

The specific gRNA sequences were selected using the CRISPR Design Tool (http://crispr.mit.edu/) or E-CRISP (http://e-crisp-test.dkfz.de/E-CRISP/) and cloned into the gRNA/Cas9 dual expression vector pSpCas9(BB)-2A-GFP or pSpCas9(BB)-2A-Puro^45^. After transfection, single cells were selected by puromycin resistance or sorted using the GFP signal by fluorescence-activated cell sorting using an S3e cell sorter (Bio-Rad) or JSAN desktop cell sorter (Bay Bioscience) and grown to confluence. The resulting clones were sequenced for verification using an ABI 3130 sequencer (Applied Biosystems).

### Real-time RT-qPCR analysis

Total RNA was subjected to RT using RiverTra Ace qPCR RT Master Mix with a gDNA Remover kit (TOYOBO Life Science) according to the manufacturer’s protocol. Real-time PCR was performed using the StepOnePlus Real-Time PCR System (Applied Biosystems) with Power SYBR Green PCR Master Mix (Applied Biosystems). The primer sequences were as follows: mHRI.SP1: 5′-AACCCGCTCCACTCCAAACA-3′; mHRI.AP1: 5′-CTCTGTTGTGGTGGAGTCTCA-3′; mActinβ.SP1: 5′-CTAAGGCCAACCGTGAAAAG-3′ and mActinβ.AP1: 5′-ACCAGAGGCATACAGGGACA-3′.

### Immunoblot analysis

Cells were lysed in TNT buffer (50 mM Tris, pH 7.5, 150 mM NaCl, 10% glycerol, 1% Triton X-100) with protease inhibitor cocktail (Nacalai Tesque), phosphatase inhibitor cocktail (Biotool), and 10 μM MG132 for standard SDS-PAGE or Phos-tag SDS-PAGE. Immunoblot analysis was performed as previously described using Blocking One (Nacalai Tesque) or Blocking One-P (Nacalai Tesque) and WesternSure ECL Substrate (Li-Cor Biosciences)^46^. Protein was visualized by Ez-Capture II (ATTO Corp) and the band intensities were quantified using Image Studio software (Li-Cor Biosciences). The sources of antibodies were as follows: Phospho-Ser51-eIF2α (D9G8) (Cell Signaling Technology); eIF2α (D7D3) (Cell Signaling Technology); anti-PERK (C33E10) (Cell Signaling Technology); anti-GCN2 (Cell Signaling Technology); anti-PKR (M-515) (Santa Cruz Biotechnology); anti-FLAG M2 (Sigma Aldrich); anti-HRI (sc-30143) (Santa Cruz Biotechnology) and anti-HRI (ab84980) (Abcam).

### Phylogenetic analysis

To generate phylogenetic trees for the eIF2AK family, the amino acid sequences of the eIF2AK family were retrieved from GenBank (http://www.ncbi.nlm.nih.gov/genbank/) as listed in Table 1. The sequences were aligned using CLUSTAL W 2.1^47^. The phylogenetic trees were constructed via the neighbour-joining method using MEGA 5.05^48^. Internal branch support was estimated with 1000 bootstrap replicates.

### Statistical analysis

Statistical analysis was performed by Student’s *t*-test. Data are expressed as the mean ± SD. P < 0.05 was defined as the threshold of significance, unless otherwise stated.

## Additional Information

**How to cite this article**: Taniuchi, S. *et al*. Integrated stress response of vertebrates is regulated by four eIF2α kinases. *Sci. Rep.* **6**, 32886; doi: 10.1038/srep32886 (2016).

## Supplementary Material

Supplementary Information

## Figures and Tables

**Table 1:** Accession numbers and names of eIF2α kinases for alignment and phylogenetic analysis.

Organism	Name	Description	GenBank Accession No
*Hammondia hammondi*	HhIF2K-A	eIF2 kinase IF2K-A	KEP59836
*Hammondia hammondi*	HhIF2K-B	eIF2 kinase IF2K-B	KEP63559
*Hammondia hammondi*	HhIF2K-C	eIF2 kinase IF2K-C	KEP61908
*Hammondia hammondi*	HhIF2K-D	eIF2 kinase IF2K-D	KEP64076
*Toxoplasma gondii*	TgIF2K-A	eIF2 kinase IF2K-A	KFH03051
*Toxoplasma gondii*	TgIF2K-B	eIF2 kinase IF2K-B	KFH09520
*Toxoplasma gondii*	TgIF2K-C	eIF2 kinase IF2K-C	KFH07289
*Toxoplasma gondii*	TgIF2K-D	eIF2 kinase IF2K-D	KFH04004
*Arabidopsis thaliana*	AtGCN2	GCN2 homologue	CAD30860
*Schizosaccharomyces pombe*	SpHRI1p	eIF2 kinase Hri1p	AAN04053
*Schizosaccharomyces pombe*	SpHRI2p	eIF2 kinase Hri2p	AAN04054
*Schizosaccharomyces pombe*	SpGCN2	Gcn2	AAU11313
*Caenorhabditis elegans*	CePEK	eukaryotic translation initiation factor 2 α kinase PEK	AAF61201
*Caenorhabditis elegans*	CeGCN2	eukaryotic translation initiation factor 2-α kinase gcn-2	D0Z5N4
*Drosophila melanogaster*	DmPEK	pancreatic eIF-2α kinase, isoform A	NP_649538
*Drosophila melanogaster*	DmGCN2	Gcn2, isoform A	NP_477230
*Danio rerio*	DrHRI	eukaryotic translation initiation factor 2-α kinase 1	NP_001071035
*Danio rerio*	DrPKR	interferon-induced double-stranded RNA-activated protein kinase	NP_001107942
*Danio rerio*	DrPERK	Eukaryotic translation initiation factor 2-α kinase 3	AAI22105
*Danio rerio*	DrGCN2	GCN2 protein, partial	CAR66088
*Mus musculus*	MmHRI	eukaryotic translation initiation factor 2-α kinase 1	NP_038585
*Mus musculus*	MmPKR	interferon-induced double-stranded RNA-activated protein kinase	NP_035293
*Mus musculus*	MmPERK	eukaryotic translation initiation factor 2-α kinase 3 isoform 1 precursor	NP_034251
*Mus musculus*	MmGCN2	eukaryotic translation initiation factor 2-α kinase 4 isoform 1	NP_038747
*Homo sapiens*	HsHRI	eukaryotic translation initiation factor 2-alpha kinase 1 isoform a	NP_055228
*Homo sapiens*	HsPKR	interferon-induced, double-stranded RNA-activated protein kinase isoform a	NP_001129123
*Homo sapiens*	HsPERK	eukaryotic translation initiation factor 2-alpha kinase 3 isoform 1 precursor	NP_004827
*Homo sapiens*	HsGCN2	eukaryotic translation initiation factor 2-alpha kinase 4	NP_001013725
