# Supplementary material for: Integrated stress response of vertebrates is regulated by four eIF2α kinases
Source: Sci Rep. 2016 Sep 16;6:32886. doi: 10.1038/srep32886 (PMC5025754; doi:10.1038/srep32886)
Supplement: Supplementary Information [file srep32886-s1.pdf]

Integrated stress response of vertebrates is regulated by four eIF2α kinases

Shusuke Taniuchi[1,2,3] Masato Miyake[1,2,3], Kazue Tsugawa[1,2], Miho Oyadomari[1,2] & Seiichi Oyadomari[1,2,3]*

Affiliations

[1]Division of Molecular Biology, Institute for Genome Research, Institute of Advanced Medical Sciences, Tokushima University, Tokushima 770-8503, Japan

[2]Department of Molecular Research, Diabetes Therapeutics and Research Center, Institute of Advanced Medical Sciences, Tokushima University, Tokushima 770-8503, Japan

[3]Fujii Memorial Institute of Medical Sciences, Institute of Advanced Medical Sciences, Tokushima University, Tokushima 770-8503, Japan

***Corresponding author:**

Seiichi Oyadomari, M.D., Ph.D.

Professor and Chairman

Institute of Advanced Medical Sciences, Tokushima University, Tokushima 770-8503, Japan

E-mail: oyadomar@tokushima-u.ac.jp, Phone: +81-88-633-9450, Fax: +81-88-633-9492

Additional information

Competing financial interests

The authors have no competing interests as defined by Nature Publishing Group, or other interests that might be perceived to influence the results and/or discussion reported in this paper.

Supplementary Information (Figures)

Supplemental Figure S1. Validation of protein phosphorylation of PKR

Supplemental Figure S2. Sequence information of *Hri gene* editing

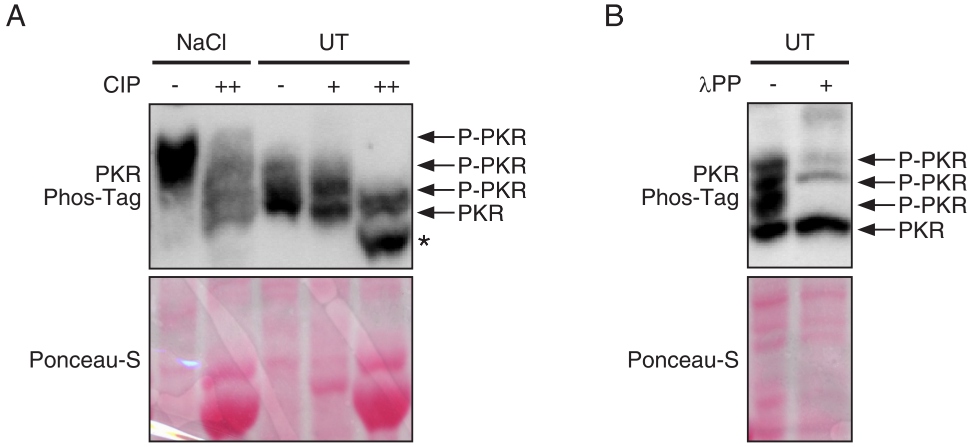

Supplemental Figure 1. Validation of protein phosphorylation of PKR

(A) Representative immunoblots of phosphorylated PKR in the presence and absence of calf intestinal phosphatase (CIP). Proteins were extracted from the wild-type MEFs 1 h after treatment with mock (UT) or 500 mM NaCl (NaCl). Ponceau S staining served as a loading control. (B) Representative immunoblots of phosphorylated PKR in the presence and absence of Lambda protein phosphatase (λPP). Proteins were extracted from the wild-type MEFs and Ponceau S staining served as a loading control.

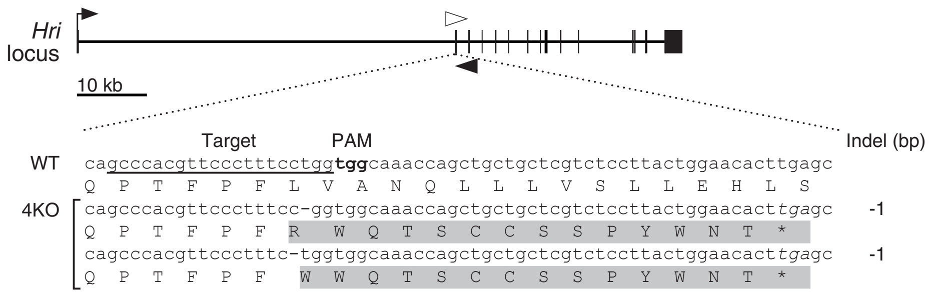

Supplemental Figure 2. Sequence information of *Hri gene* editing

Schematic of targeting locus of HRI with the target sequence underscored, PAM in bold type and exons in block boxes. Predicted consequences of the mutation on the amino acid sequences are highlighted. Stop codons are shown as *.
